# A Case of Respiratory Syncytial Virus Infection in an HIV-Positive Adult

**DOI:** 10.1155/2012/267028

**Published:** 2012-09-12

**Authors:** Aakriti Gupta, Purav Mody, Shefali Gupta

**Affiliations:** ^1^Center for Outcomes Research and Evaluation, Yale University, New Haven, CT 06510, USA; ^2^Department of Microbiology, Kasturba Medical College, Mangalore 575001, India

## Abstract

Respiratory syncytial virus (RSV) is commonly known to cause an influenza-like illness. However, it can also cause more severe disease in young children and older adults comprising of organ transplant patients with immunocompromised status. Till date, only four cases of RSV infections have been reported in HIV-positive adults. We describe here a case of HIV-positive female with relatively preserved immune function who presented with RSV infection requiring ventilation and showed improvement after prompt treatment with intravenous immunoglobulin.

## 1. Introduction

Respiratory syncytial virus (RSV) is a common virus widely known to cause acute respiratory tract illness in people of all ages. Young immunocompetent children are more frequently infected, and reinfection is common. However, it has come to be recognized as a serious adult pathogen in recent times. Epidemiological studies indicate that RSV is second to influenza as a cause of serious viral respiratory disease in adults [1].

Immunocompromised adults may have RSV in varying degrees of severity and outcomes ranging from full recovery to progressive respiratory failure and death. Most case studies involving RSV infection in such patients include hematopoietic stem cell transplant (HSCT) or lung transplant patients [2]. Only four cases have been reported in HIV-positive individuals [3–6]. 

We describe here a case of 55 y/o HIV-positive female with respiratory failure on mechanical ventilation detected to have RSV infection.

## 2. Case History

We describe a case of an HIV-positive 55-year-old female who presented with worsening shortness of breath, cough and fevers for one-week duration associated with right-sided pleuritic chest pain. Her antiretroviral therapy regimen consisted of Tenofovir/Emtricitabine, Ritonavir, and Darunavir daily. Her CD4 count on admission was 408/mm^3^ with undetectable viral load. 

Patient was febrile to a maximum temperature of 102 F, hypotensive with systolic blood pressure recorded in the range of 80–90 mm Hg, and hypoxic with oxygen saturation of 90% on room air. On auscultation, she had bilateral crackles anteriorly. Chest X-ray demonstrated right-sided pleural effusion with bibasilar opacities. On admission, patient had acute renal failure with a creatinine of 4.3.

In the emergency department, patient received ceftriaxone and azithromycin as empiric coverage for community-acquired pneumonia, intravenous fluids, and bronchodilators. Her respiratory status declined overnight with worsening hypoxia requiring intubation and mechanical ventilation. Repeat chest X-ray showed evidence of worsening pleural effusions that were now bilateral. Vancomycin was added to her antibiotic regimen and azithromycin was discontinued. Upon intubation, thick-bloody, yellow-green sputum was suctioned from the airways. A bronchoalveolar lavage was performed to obtain specimens for pathology and culture.

Bacterial, fungal, viral (Influenza A and B), pneumocystis, and mycobacterial cultures remained negative over the next few days. On hospital day 3, RSV Ag was detected by ELISA in the bronchoalveolar lavage specimen collected under direct visualization. Her renal failure precluded the use of aerosolized ribavirin. Also, limited drug availability and inadequate experience of the staff personnel with its usage precluded its use. However, we administered intravenous immunoglobulin (IVIG) 35 g/day for four days as recommended. Meanwhile, we continued treatment with vancomycin and ceftriaxone. 

After 4 doses of IVIG and the above antibiotics, her oxygenation and respiratory symptoms started improving by hospital day 7. Her oxygen saturation rose to 100% on FiO_2_ of 40%, and renal function improved with creatinine decreasing to 1.4. She also regained her mental status to the extent that she would respond to some commands, though not fully.

Her clinical status markedly improved, and she was finally extubated. A follow-up bronchial alveolar lavage for the detection of RSV was ordered, and results were negative.

## 3. Discussion

RSV has been clearly recognized as a pathogen afflicting all age groups and both immunocompetent and immunocompromised people. 

Among the immunocompromised adults, most experience in treating serious RSV infections has been reported with HSCT or lung transplant recipients [2]. Information regarding RSV infection in HIV/AIDS patients and its management that exists in current literature is rather limited. To our knowledge, only four cases of HIV-positive patients with RSV infection have been reported in literature (Table 1) [3–6]. Given the lack of evidence-based data, we have to depend on a few case reports to guide the management of these patients. Hence, recognition and treatment of RSV in the immunocompromised patients, HIV-positive in particular, remains a challenge.

Detection of RSV in clinical specimens such as nasal washes or bronchoalveolar lavages can be made by various diagnostic methods including viral culture, detection of viral antigens, and detection of viral RNA. Rapid diagnosis of RSV can be made by direct antigen testing on clinical specimens (i.e., direct immunofluorescence staining), with a sensitivity of 93% and a specificity of 97%, and by real-time polymerase chain reaction (RT-PCR) assays for detection of RSV RNA with a higher sensitivity and specificity [7, 8]. RSV usually manifests as an upper respiratory tract infection but may progress rapidly to lower respiratory tract infection (LRI). Studies in the HSCT population demonstrate increased morbidity and mortality with LRI [9]. 

Our patient had a CD4 count of 409/mm^3^ on admission; hence her immune function was relatively preserved at presentation. This is in contrast to previous cases reported in literature with severe immunocompromise (Table 1). Interestingly, a substantially high number of immunocompetent adults are being identified to have RSV infection. In a four-year prospective study consisting of 608 healthy elderly patients, 540 high-risk adults, and 1388 hospitalized patients, RSV was identified in 102 patients in the prospective cohorts and in 142 hospitalized patients [10]. Among the healthy elderly cohort with RSV infection, 11% of the patients had findings on chest X-ray, and 76% of patients had functional impairment for more than one day. 

Commonly advocated therapies for RSV include aerosolized ribavirin and immunoglobulin products (intravenous immunoglobulin, RSV hyperimmune globulin, or the RSV-specific monoclonal antibody palivizumab). Some studies have suggested that dual therapy with aerosolized ribavirin and an intravenous immunoglobulin product should be the standard of care until controlled trials are available. The crucial factor, however, in the management of RSV infections is the promptness in institution of therapy. Mortality rates have been shown to be significantly higher when therapy is delayed [11].

In our patient, institution of IVIG was initiated promptly when RSV antigen was detected. She received four doses and gradually showed improvement in her respiratory status thus emphasising the importance of early detection and prompt institution of antiviral therapy.

## Figures and Tables

**Table 1:** Previous case reports of RSV in HIV-positive adults.

Study	CD4 count at presentation (cells/cu mm)	Diagnostic technique	RSV-specific management
Murphy and Rose [5]	170	Culture of bronchial washings	None
Sriskandan and Shaunak [6]	130	Immunofluorescence of bronchial lavage	Ribavirin
Voigt et al. [3]	8	RT-PCR^a^	None
Cunha et al. [4]	43	Bronchoscopy washings cytology	None
Our study	409	Bronchial lavage ELISA^b^	IVIG^c^

^a^RT-PCR: reverse transcriptase polymerase chain reaction. ^b^ELISA: enzyme linked immunosorbent assay. ^c^IVIG: intravenous immunoglobulin.
